# Short-Term High-CO_2_ Treatment Modulates Phenylpropanoid Metabolism and Antioxidant Capacity in Blueberries During Cold Storage

**DOI:** 10.3390/plants15101496

**Published:** 2026-05-14

**Authors:** Jose David Toledo-Guerrero, María Teresa Sanchez-Ballesta, Claudia Balderas, María Isabel Escribano, Carmen Merodio, Irene Romero

**Affiliations:** 1 Department of Characterization, Quality and Safety, Institute of Food Science, Technology and Nutrition (ICTAN-CSIC), Jose Antonio Novais 6, 28040 Madrid, Spain; j.toledo@ictan.csic.es (J.D.T.-G.); mballesta@ictan.csic.es (M.T.S.-B.); claudia.balderas@iqog.csic.es (C.B.); escribano@ictan.csic.es (M.I.E.); merodio@ictan.csic.es (C.M.); 2 Faculty of Biological Sciences, Complutense University of Madrid, Jose Antonio Novais 12, 28040 Madrid, Spain

**Keywords:** *Vaccinium* spp., postharvest physiology, secondary metabolism, phenolic compounds, transcriptional regulation

## Abstract

Maintaining the nutritional and functional quality of blueberries during cold storage remains a postharvest challenge. This study evaluated the effects of short-term high-CO_2_ treatments on phenylpropanoid-related metabolism in two commercially important blueberry cultivar–season combinations: Duke (*Vaccinium corymbosum* L., highbush; early-season, June harvest) and Ochlockonee (*V. virgatum* Aiton, rabbiteye; late-season, September harvest). Fruits were exposed to 15% or 20% CO_2_ for 3 days at 1 °C and subsequently stored for up to 29 days at 1 °C. Phenolic compounds, antioxidant capacity, and the expression of selected phenylpropanoid-related genes, including flavonoid biosynthetic enzymes and R2R3-MYB transcription factors, were analyzed. Short-term CO_2_ treatments were associated with transient transcriptional responses, particularly in anthocyanin-related pathways, together with genotype-associated differences in phenolic composition and antioxidant capacity during storage. Overall, the results indicate associations between CO_2_ exposure and secondary metabolism under cold storage conditions and should be interpreted as correlative rather than mechanistic evidence of priming.

## 1. Introduction

Growing awareness of the health benefits associated with blueberry (*Vaccinium* spp.) consumption has led to a notable increase in demand in recent years [1]. As a crop, blueberries are known for their high productivity, adaptability, and resistance to environmental stress [2]. The United States is the largest global producer of blueberries, followed by Canada, both of which together represent the main production base in North America. Collectively, these countries account for 63.3% of global blueberry production. In the Southern Hemisphere, Chile and Peru have become key producing and exporting countries, contributing an additional 20.5% of global production, driven by the recent rapid expansion of the industry. In Europe, blueberry production remains more fragmented, with Poland, Germany and Portugal among the main producers, while Spain represents one of the most important producers in Southern Europe due to its early season and export capacity [3].

This rapid expansion of global production has been accompanied by increasing logistical and postharvest challenges, as blueberries are highly perishable and their quality can deteriorate rapidly during storage and transport. These losses directly impact commercial value, storage performance, and distribution efficiency along the supply chain, making postharvest quality preservation a critical issue for the industry [4].

Blueberries are rich in secondary metabolites, particularly anthocyanins and other polyphenols, which contribute to their appearance, color and flavor [1]. Moreover, these phytochemicals exhibit potent antioxidant activity and are widely recognized for their role in the prevention of various chronic human diseases [5]. In addition, these compounds also perform critical physiological roles by influencing plant growth and mediating interactions with the environment. They are particularly involved in responses to abiotic stress and in defense mechanisms against pathogens [6]. Beyond their nutritional and functional properties, phenolic compounds and anthocyanins play a critical role in determining key commercial quality attributes of blueberries, including skin color, visual appeal, and resistance to postharvest deterioration. These attributes directly influence consumer acceptance, marketability, and shelf life. During cold storage, the degradation or insufficient accumulation of these compounds can lead to color loss, reduced firmness, and increased susceptibility to decay, ultimately limiting commercial value [7,8]. Therefore, strategies aimed at preserving or enhancing phenolic metabolism during postharvest storage are not only relevant from a nutritional standpoint but also essential for maintaining fruit quality and extending shelf life.

Importantly, fruit quality and postharvest performance are not only determined during storage but are largely established during cultivation, as preharvest conditions shape the metabolic composition that is subsequently maintained or modified after harvest. Understanding how postharvest treatments interact with this pre-established biochemical background is therefore essential for interpreting changes in fruit quality [9].

The phenylpropanoid pathway is a central biosynthetic pathway that produces secondary metabolites such as flavonoids, lignans, and phenolic acids [10]. Central enzymes like phenylalanine ammonia-lyase (PAL), cinnamate 4-hydroxylase (C4H), and 4-coumarate:CoA ligase (4CL) initiate the pathway by converting phenylalanine into p-coumaroyl-CoA, a precursor for multiple branches, including flavonoid and anthocyanin biosynthesis [11]. Anthocyanin formation involves chalcone synthase (CHS), chalcone isomerase (CHI), flavonol synthase (FLS), and UDP-glucose:flavonoid 3-O-glucosyltransferase (UFGT), resulting in compounds like quercetin, myricetin, and various anthocyanins [12,13]. Regulation of this pathway is largely controlled by MYB (Myeloblastosis) transcription factors, which can act as activators or repressors at different stages [14]. For example, in *Arabidopsis thaliana*, *MYB21*, an R2R3-MYB transcription factor, promotes flavonol synthesis by regulating the expression of the *FLS* gene [15].

The levels of phenolic compounds in soft fruits such as blueberries are influenced by genetic background, harvest maturity, and postharvest conditions [16,17]. The accumulation of anthocyanins and total phenolics during cold storage is cultivar-dependent and often associated with increased antioxidant capacity [18,19].

Cold storage is one of the most widely used methods for preserving blueberries; however, it can lead to several quality-related issues, including fungal attacks, primarily caused by *Botrytis cinerea*, as well as fruit softening, peduncle splitting, and adhesion of the pericarp to the pulp [17,20]. To mitigate these issues, controlled atmosphere (CA) and modified atmosphere (MA) techniques have been applied, typically involving prolonged exposure to reduced O_2_ and/or elevated CO_2_ levels throughout storage. These approaches help extend shelf life by slowing down metabolic activity and delaying senescence [21]. In contrast, short-term high-CO_2_ treatments consist of brief exposure (e.g., 1–3 days) to elevated CO_2_ concentrations before storage under atmospheric conditions. Unlike continuous CA or MA systems, which maintain altered gas levels throughout storage, these short-term treatments act as abiotic stressors that may be associated with physiological and molecular changes, such as modulation of gene expression and secondary metabolism. These short-term high-CO_2_ treatments have been reported to be effective in prolonging the postharvest shelf life of blueberries and other soft fruits, including raspberries and strawberries [22,23].

In a previous study [24], it was reported that short-term gaseous treatments with 15% or 20% CO_2_ for 3 days effectively reduced total decay in both highbush (cv. Duke) and rabbiteye (cv. Ochlockonee) blueberries during cold storage. However, their effectiveness in preserving firmness and minimizing weight loss was genotype-associated, as improvements in mechanical properties were observed only in highbush blueberries, while reduced weight loss was found in rabbiteye blueberries. It is important to emphasize that these two cultivars represent distinct genetic backgrounds and harvest maturities, and therefore constitute model systems rather than fully controlled species-level comparisons.

Despite these promising results, the impact of such gaseous treatments on secondary metabolite accumulation and their transcriptional regulation in blueberries remains largely unexplored. Given the importance of these compounds for fruit quality, it is important to characterize how short-term CO_2_ treatments are associated with changes in metabolite accumulation and gene expression across different blueberry cultivars.

Accordingly, this study evaluated the effects of short-term CO_2_ treatments (15% and 20% for 3 days at 1 °C) on anthocyanins and phenolic accumulation in highbush (cv. Duke) and rabbiteye (cv. Ochlockonee) blueberries during cold storage. Total phenolics, anthocyanin content, antioxidant capacity, phenolic profiles, and expression of phenylpropanoid-related genes, including key MYB transcription factors, were analyzed over 29 days of storage.

The study provides a correlative analysis of transcriptional and metabolic responses to short-term high-CO_2_ exposure in two genetically distinct blueberry cultivars, without aiming to infer causal mechanisms.

## 2. Material and Methods

### 2.1. Plant Material and Postharvest Treatments

Two commercially important blueberry cultivars belonging to different species were selected: Duke (*Vaccinium corymbosum* L., northern highbush) and Ochlockonee (*V. virgatum Aiton*, rabbiteye). Fruit were harvested from certified organic orchards managed under standard commercial practices. Fruits were collected by the grower from multiple plants within each orchard and pooled into commercial batches, following standard fresh-market practices; therefore, orchard- and plant-level replication was not defined in the experimental design. These cultivars are widely cultivated and differ in harvest time and postharvest behavior, with Duke representing an early-season cultivar (June) and the other a late-season cultivar (September), and were selected as contrasting genotypes for evaluating differential responses to short-term high-CO_2_ treatments.

Fruits were harvested at the commercial ripening stage defined by the grower, corresponding to market-ready fruit characterized by full skin coloration and suitability for fresh consumption [25]. This maturity stage was selected to ensure comparability in terms of commercial readiness and postharvest handling conditions, as commonly applied in industrial supply chains, while acknowledging inherent genotype-related differences in compositional parameters as part of the experimental design. Accordingly, Duke presented SSC (°Brix): 9.17; TA (% citric acid): 0.87; pH: 2.82, whereas Ochlockonee showed SSC (°Brix): 12.67; TA (% citric acid): 0.34; and pH: 2.81. Harvest took place during their respective seasons, June (Duke) and September (Ochlockonee) 2022, in the Salas village of Asturias, Spain (43°24′35″ N, 6°15′38″ W; 243 m altitude).

Uniform and disease-free blueberries from both cultivars were manually harvested and placed in 125 g eco-friendly solid board punnets (Smurfit Kappa Polska, Warszawa, Poland) equipped with a lid containing a transparent cellulose film window (Futamura Chemical Co., Ltd., Nagoya, Japan). The containers (145 × 85 × 50 mm; 16 g) allowed adequate ventilation and handling during storage. Following harvest, fruits were transported to the laboratory facilities (ICTAN) on the same day. Upon arrival, punnets were randomly assigned to three experimental groups (21 punnets per group), and treatments were applied immediately. The fruits were then stored in 1 m^3^ methacrylate chambers at 1.0 ± 0.5 °C and 95% relative humidity. A subset of three randomly selected punnets was immediately analyzed and considered as time 0. One group was maintained under normal atmospheric conditions (non-treated fruit) for 29 days. The remaining two groups were subjected to short-term gaseous treatments consisting of either 15 kPa CO_2_ + 20 kPa O_2_ + 65 kPa N_2_ (15% CO_2_-treated fruit) or 20 kPa CO_2_ + 20 kPa O_2_ + 60 kPa N_2_ (20% CO_2_-treated fruit) for 3 days at 1 °C. After this treatment period, fruits were transferred to air storage under the same temperature and humidity conditions as the control for the remaining 26 days.

Fruit samples were collected at four time points (0, 3, 14, and 29 days) to monitor both the immediate and long-term effects of the postharvest treatments. For each sampling point, fruits were randomly selected from different punnets within each treatment group to ensure representative sampling of the population and minimize potential container-related bias. This sampling strategy ensured that biological replication reflected commercial batch variability rather than within-punnet variation. After collection, whole fruits (including skin and flesh) were immediately frozen in liquid nitrogen, ground into a fine powder, and stored at −80 °C until further analyses.

### 2.2. Relative Gene Expression by Quantitative Real-Time RT-PCR (RT-qPCR)

Total RNA was isolated from 0.4 g of powdered frozen whole blueberry tissue (including skin and flesh), obtained from pooled fruit samples, using three biological replicates. Each biological replicate consisted of a composite sample of multiple blueberries collected from different punnets within the same treatment group, in order to reduce intra-sample variability. RNA extraction was performed following the method described by [26], with slight modifications.

First-strand cDNA was synthesized from 0.7 µg of total RNA using the Maxima First Strand cDNA Synthesis Kit with dsDNase (Thermo Fisher Scientific, Waltham, MA, USA), as per the manufacturer’s protocol. RT-qPCR was carried out using an iCycler iQ thermal cycler (Bio-Rad Laboratories, Hercules, CA, USA), and transcript quantification was performed with Real-Time Detection System Software (version 2.0), following the methodology outlined by [24].

Gene sequences used for expression profiling were obtained from the Blueberry Genome Database (Genome Data Base for *Vaccinium*, GDV; *Vaccinium corymbosum* cv. Draper v1.0 genome sequence, https://www.vaccinium.org/). The nomenclature of *MYB* genes in this study follows the most recent comprehensive annotation of R2R3-MYB family genes in *V. corymbosum* [27]. Primer design for target genes (listed in Supplementary Table S1) was carried out using Primer3 software [28]. Primer specificity was experimentally validated in both cultivars by agarose gel electrophoresis, confirming single amplicons of the expected size. In addition, selected amplicons were purified and sequenced using Sanger sequencing at Secugen S. L. (Madrid, Spain) to confirm their identity.

Gene expression levels were calculated using the 2^−ΔΔCT^ method [29], with elongation factor 1-alpha (*EF1α*) as the internal reference gene and the day 0 sample as the calibrator [24,30]. Each reaction was performed with three biological replicates and two technical replicates per gene.

### 2.3. Total Phenolic and Total Anthocyanin Content

Phenolic and anthocyanin extracts were obtained as follows: 0.2 g of frozen powdered fruit tissue was homogenized with 1 mL of a methanol–water solution (50:50, *v*/*v*) acidified with 1% HCl. The mixture was incubated under agitation for 1 h at room temperature, protected from light, and subsequently centrifuged at 10,000× *g* for 10 min. The supernatant was collected and kept on ice while the extraction was repeated on the pellet under the same conditions. Both supernatants were combined to a final volume of 2 mL, filtered through 0.45 µm nylon filters, and stored at −20 °C until analysis.

Total phenolic content (TPC) was assessed using a slightly modified version of the Folin–Ciocalteu method [31]. Briefly, 25 µL of the extract was mixed with 25 µL Folin–Ciocalteu reagent and pre-incubated for 5 min at room temperature in the dark. Subsequently, 500 µL of sodium carbonate solution (75 g L^−1^) was added and the mixture was incubated for 1 h in darkness before measuring absorbance at 765 nm. Absorbance values were converted into gallic acid equivalents using a calibration curve (0–5 mg mL^−1^). The results were expressed as mg gallic acid equivalents (GAE) × 100 g^−1^ fresh weight (FW).

Total anthocyanin content (TAC) was determined using the pH differential method [32], with adaptations as described by [33]. Briefly, the extracts were diluted 1:10 by mixing 100 µL of extract with 900 µL of potassium chloride buffer (25 mM KCl, pH 1.0) or with 900 µL of sodium acetate buffer (0.4 M sodium acetate, pH 4.5). Absorbance was measured at 520 and 700 nm in potassium chloride buffer (pH 1.0) and sodium acetate buffer (pH 4.5), and anthocyanin concentration was calculated according to

Anthocyanin content (mg L^−1^) = (A × MW × DF × 1000)/(ε × l)

where A = (A_520_ − A_700_) pH 1.0 − (A_520_ − A_700_) pH 4.5, MW is the molecular weight of cyanidin-3-glucoside (449.2 g mol^−1^), DF is the dilution factor, ε is the molar extinction coefficient (26,900 L mol^−1^ cm^−1^), and l is the path length (1 cm). Results were expressed as mg cyanidin-3-glucoside equivalents × 100 g^−1^ FW.

All measurements were performed using three biological replicates per sample, each with two technical replicates.

### 2.4. Antioxidant Activity (ABTS and FRAP)

Antioxidant activity was evaluated using two complementary assays: the ABTS^+^ radical cation decolorization method [34] and the ferric reducing antioxidant power (FRAP) assay [35]. Both analyses were conducted using the same extracts previously prepared for phenolic and anthocyanin quantification. Trolox was used as the standard antioxidant to generate a calibration curve within the 0–4 mM range. Results were expressed as µmol Trolox Equivalents (TE) × 100 g^−1^ FW. For the ABTS assay, the ABTS^+^ radical cation was generated by mixing 7 mM ABTS and 2.45 mM potassium persulfate in a 2:1 ratio and incubating the mixture for 16 h in darkness. Before use, the ABTS^+^ solution was diluted to obtain an absorbance of 0.68–0.72 at 734 nm. The assay mixture contained 5 µL of extract, 5 µL of distilled water, and 1 mL of diluted ABTS^+^ solution, and absorbance was monitored at 734 nm at 30 °C after reaction with the radical solution.

For the FRAP assay, the working reagent was freshly prepared by mixing 10 mM TPTZ in 40 mM HCl, 20 mM FeCl_3_·6H_2_O, and acetate buffer (sodium acetate/acetic acid buffer) in a 1:1:20 ratio. The reaction mixture consisted of 5 µL of extract, 115 µL of distilled water and 900 µL of FRAP reagent, followed by incubation for 30 min at 37 °C before absorbance measurement at 593 nm.

Each assay included three independent biological replicates per sample, with two technical replicates per extract to confirm analytical repeatability, as these spectrophotometric methods show low instrumental variability.

### 2.5. Identification and Quantification of Phenolic Compounds Using HPLC-QTOF

Aliquots of the extracted phenolic compounds were analyzed using high-performance liquid chromatography coupled to quadrupole time-of-flight mass spectrometry (HPLC-QTOF), following the method described by [36]. Analyses were performed in both positive and negative electrospray ionization modes. Three biological replicates per treatment were analyzed; technical replicates were not performed, as HPLC–QTOF provides high instrumental reproducibility under controlled analytical conditions, and the study aimed to capture biologically relevant variability among samples rather than injection repeatability. The quality of chromatographic runs was evaluated through preliminary inspection of TICs using Agilent MassHunter Qualitative Analysis (version B.07.00). Subsequently, raw data were processed with Agilent MassHunter Profinder (version B.10.00) using the Batch Targeted Feature Extraction workflow. This process allowed peak alignment, noise reduction, and grouping of correlated ions for targeted metabolite analysis.

Identification of phenolic compounds was carried out using commercial standards, when available. These included epicatechin (C_15_H_14_O_6_), chlorogenic acid (C_16_H_18_O_9_), gallic acid (C_7_H_6_O_5_), coumaric acid (C_9_H_8_O_3_), caffeic acid (C_9_H_8_O_4_), and quercetin-3-glucoside (C_21_H_20_O_12_), as well as anthocyanins such as malvidin-3-glucoside (C_23_H_25_O_12_), cyanidin-3-glucoside (C_21_H_21_O_11_), cyanidin-3-rutinoside (C_27_H_31_O_15_), delphinidin-3-rutinoside (C_27_H_31_O_16_), and pelargonidin-3-glucoside (C_21_H_21_O_10_). Standard solutions were prepared within a concentration range of 0.1–100 ppm. Linearity was confirmed for all quantified compounds, with correlation coefficients (R^2^) above 0.995. Sensitivity parameters were assessed by determining LOD and LOQ, based on S/N ratios of 3:1 and 10:1, respectively. When commercial standards were unavailable, metabolite identification was based on accurate mass, retention time, isotopic pattern, and MS/MS fragmentation data, supported by comparisons with public databases such as HMDB (https://hmdb.ca/, accessed on 3 October 2024) and FooDB (https://foodb.ca/, accessed on 3 October 2024), as well as previously reported fragmentation patterns in the literature. Data analysis and metabolite annotation were conducted using MassHunter Qualitative Analysis software, following the approach described by [27]. Identification confidence levels were assigned according to the Metabolomics Standards Initiative (MSI) guidelines [37]. Compounds confirmed with authentic standards were classified as MSI level 1, whereas putatively annotated metabolites based on MS/MS data were assigned to MSI level 2. Compounds that could not be unequivocally identified and were reported as unresolved isomers were classified as MSI level 3.

### 2.6. Statistical Analyses

All statistical analyses were performed using IBM SPSS Statistics software (version 29.0; IBM Corp., Armonk, NY, USA). Data were subjected to one-way analysis of variance (ANOVA) to evaluate treatment effects at each sampling point, and mean comparisons were conducted using Tukey’s post hoc test with a significance level of *p* < 0.05. ANOVA assumptions were considered to be reasonably met for the analyzed variables.

Two-way ANOVA was performed separately for each cultivar to evaluate the effects of storage time, CO_2_ treatment, and their interaction (treatment × time). Genotype was not included as a factor in the statistical model; therefore, differences between cultivars are reported as descriptive genotype-associated responses rather than statistically tested interactions.

Pearson correlation analysis (*p* < 0.01 or *p* < 0.05) was performed to examine associations among biochemical traits, individual metabolites, and relative gene expression levels.

Principal component analysis (PCA) was carried out in SPSS using the correlation matrix of the quantitative variables, with data standardized prior to analysis. The first two principal components (PC1 and PC2) were retained for graphical representation of variable loadings.

Metabolomic variables were preprocessed using log transformation followed by Pareto scaling to reduce heteroscedasticity and improve comparability across features. Differences among experimental groups were evaluated using ANOVA. To control for multiple testing, *p*-values were adjusted using the Benjamini–Hochberg false discovery rate (FDR) method. Features with adjusted *p*-values < 0.05 were considered statistically significant.

Significant features were subsequently used to generate a heatmap to visualize their relative abundance patterns across groups. Hierarchical clustering analysis was performed in MetaboAnalyst 6.0 (https://www.metaboanalyst.ca/, accessed on 7 October 2024) using Euclidean distance and Ward’s linkage method.

## 3. Results

### 3.1. Effect of Short-Term CO_2_ Treatments on Phenylpropanoid Pathway Gene Expression in Highbush and Rabbiteye Blueberries

Key structural genes of the phenylpropanoid biosynthetic pathway were selected for expression analysis, including general pathway genes (*PAL*, *CHS*), anthocyanin-specific genes (*DFR*, *LDOX*, *UFGT*, *UGT75C1*), flavan-3-ols (*LAR*, *ANR*), flavonols (*FLS*), and *MYB246* and *MYB336* transcription factors.

**Figure 1:**
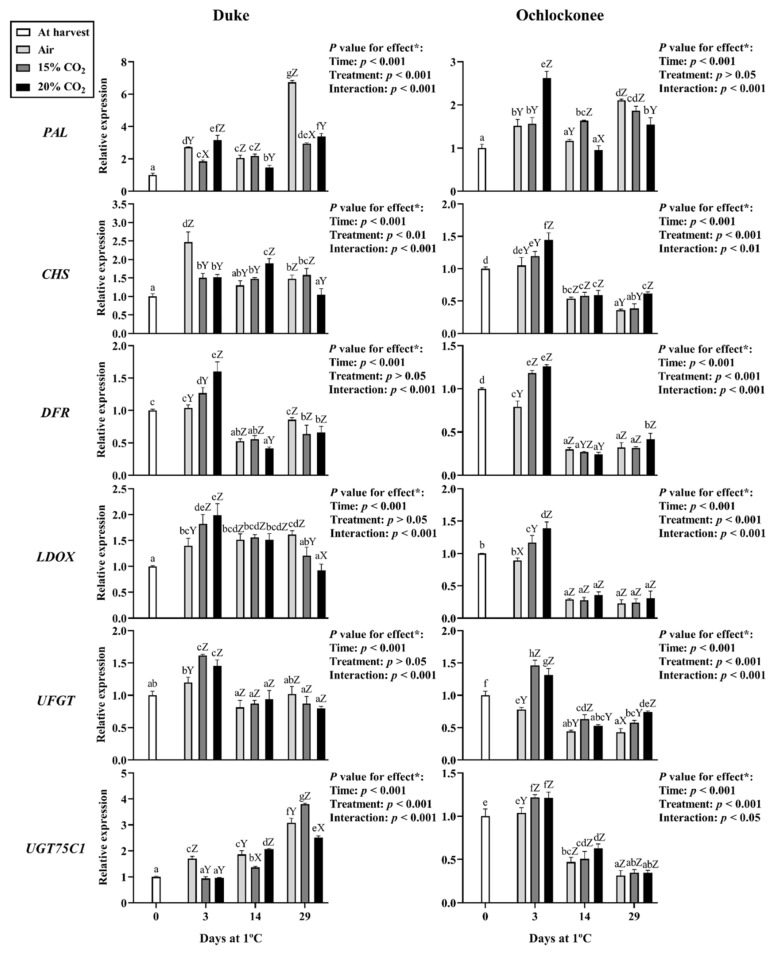
Expression of phenylpropanoid-related genes in Duke and Ochlockonee blueberries under short-term CO_2_ treatments during cold storage. Samples at day 0 are indicated as “at harvest”, while non-treated fruits are indicated as “air”. Transcript levels were determined by RT-qPCR, normalized against *EF1*, and expressed relative to day 0 using the 2^−ΔΔCt^ method. Values represent mean ± SD (*n* = 6). Different lowercase letters indicate significant differences among all groups, and uppercase letters indicate differences among treatments within the same day (Tukey’s test, *p* < 0.05). (*) *p*-values for storage time, treatment, and their interaction are shown in each panel.

At the end of the 3-day CO_2_ treatments, both highbush (Duke) and rabbiteye (Ochlockonee) blueberries exhibited changes in phenylpropanoid and anthocyanin pathway gene expression, with more pronounced responses generally observed under 20% CO_2_ (Figure 1).

Regarding *PAL* expression, at day 3, the highest transcript levels were observed in fruit from both cultivars treated with 20% CO_2_. However, by the end of storage, treated fruit showed lower expression levels than non-treated fruit in both cultivars but remained higher than at harvest (Figure 1).

*CHS* expression differed between Duke and Ochlockonee (Figure 1). In Duke, a transient increase was observed at day 14 in fruit exposed to 20% CO_2_. In Ochlockonee, *CHS* induction occurred earlier, at day 3, in fruit exposed to 20% CO_2._ In subsequent sampling points, expression generally declined in all treatments compared with day 0.

The anthocyanin biosynthetic genes *DFR*, *LDOX*, and *UFGT* showed increased transcript levels at day 3 in response to both CO_2_ treatments in the two cultivars, with generally higher values under 20% CO_2_ (Figure 1). Transcript levels subsequently decreased during storage, reaching values lower than those observed at harvest. However, in Duke, *LDOX* expression remained relatively higher throughout storage compared with freshly harvested fruit. *UGT75C1* showed genotype-associated patterns: in Duke, expression increased progressively during storage, with a significant increase observed in fruit treated with 15% CO_2_ at day 29, whereas in Ochlockonee it followed a transient induction at day 3 in CO_2_-treated fruit, followed by a decrease during storage (Figure 1).

**Figure 2:**
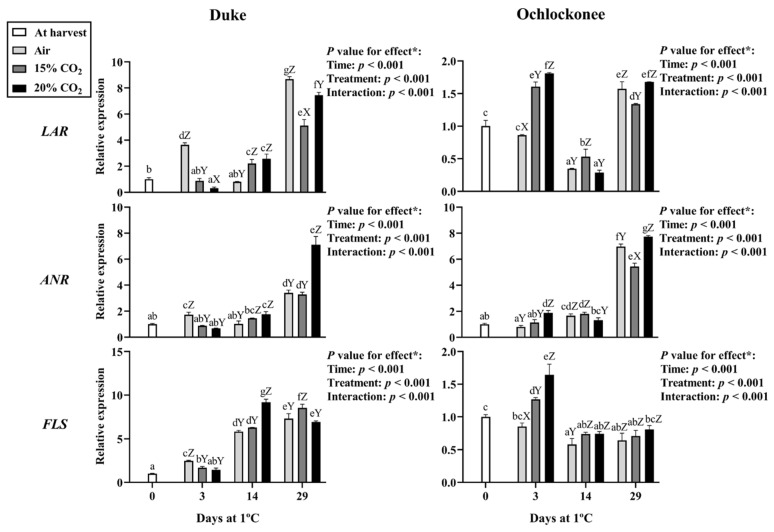
Expression of flavan-3-ol and flavonol biosynthetic genes in Duke and Ochlockonee blueberries under short-term CO_2_ treatments during cold storage. Samples at day 0 are indicated as “at harvest”, while non-treated samples stored under normal atmospheric conditions are indicated as “air”. Transcript levels were determined by RT-qPCR, normalized against *EF1*, and expressed relative to day 0 using the 2^−ΔΔCt^ method. Values represent mean ± SD (*n* = 6). Different lowercase letters indicate significant differences among all groups, and uppercase letters indicate significant differences among treatments within the same day (Tukey’s test, *p* < 0.05). (*) *p*-values for storage time, treatment, and their interaction are shown in each panel.

The flavan-3-ol-related genes *LAR* and *ANR* also showed distinct temporal and treatment-dependent patterns (Figure 2). In Duke, *LAR* expression increased at day 3 in non-treated fruit and showed a delayed increase at day 14 in CO_2_-treated fruit. At day 29, expression increased again, with higher levels observed in non-treated fruit. *ANR* expression in Duke showed an early increase in non-treated fruit at day 3, followed by higher values at later storage stages (days 14–29) in CO_2_-treated fruit, particularly under 20% CO_2_. In Ochlockonee, both *LAR* and *ANR* were induced at day 3 by CO_2_ treatments, with significant effects observed only for *ANR* under 20% CO_2_, and showed variable expression patterns during storage, with higher transcript levels again detected at day 29.

*FLS* expression in Duke increased progressively during storage, with a significant effect of 20% CO_2_ observed at day 14, whereas at day 29, the highest values were detected in fruit treated with 15% CO_2_. In Ochlockonee, *FLS* expression showed a transient increase at day 3 only in CO_2_-treated fruit and remained generally below harvest levels thereafter, with no consistent differences among treatments (Figure 2).

**Figure 3:**
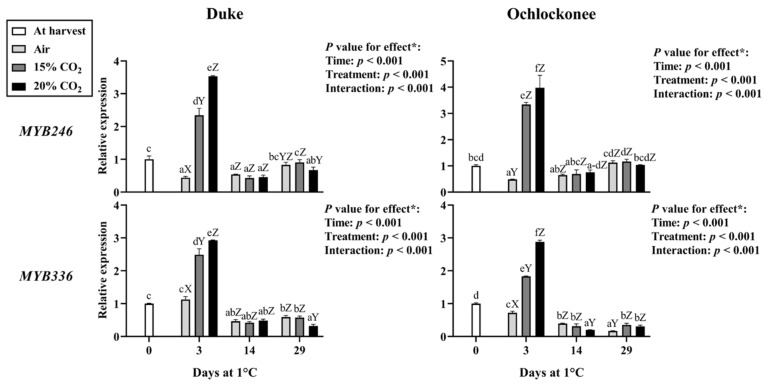
Expression of *MYB246* and *MYB336* transcription factors in Duke and Ochlockonee blueberries under short-term CO_2_ treatments during cold storage. Samples at day 0 are indicated as “at harvest”, while non-treated samples stored under normal atmospheric conditions are indicated as “air”. Transcript levels were determined by RT-qPCR, normalized against *EF1*, and expressed relative to day 0 using the 2^−ΔΔCt^ method. Values represent mean ± SD (*n* = 6). Different lowercase letters indicate significant differences among all groups, and uppercase letters indicate significant differences among treatments within the same day (Tukey’s test, *p* < 0.05). (*) *p*-values for storage time, treatment, and their interaction are shown in each panel.

The analyzed R2R3-MYB transcription factors showed a transient increase in transcript levels at day 3 in response to both CO_2_ treatments, with generally higher values under 20% CO_2_, followed by a decline during storage to levels similar to or lower than those observed in non-treated fruit (Figure 3).

Correlation analysis revealed a higher number of significant positive associations among gene expression levels in Ochlockonee compared with Duke (Supplementary Tables S2 and S3). In Duke, *PAL* expression showed significant positive correlations with *UGT75* (*p* < 0.01), *LAR* (*p* < 0.01), and *ANR* (*p* < 0.05). The anthocyanin-related genes *DFR*, *LDOX*, and *UFGT* showed positive intercorrelations and were also significantly associated with *MYB246* and *MYB336* transcription factors (*p* < 0.01). In Ochlockonee, several genes showed positive correlations; however, the strength and significance of these associations varied across gene pairs, and fewer consistent relationships were observed for *PAL* and *ANR*.

### 3.2. Effect of Short-Term CO_2_ Treatment on the Total Phenolic and Anthocyanin Content

**Figure 4:**
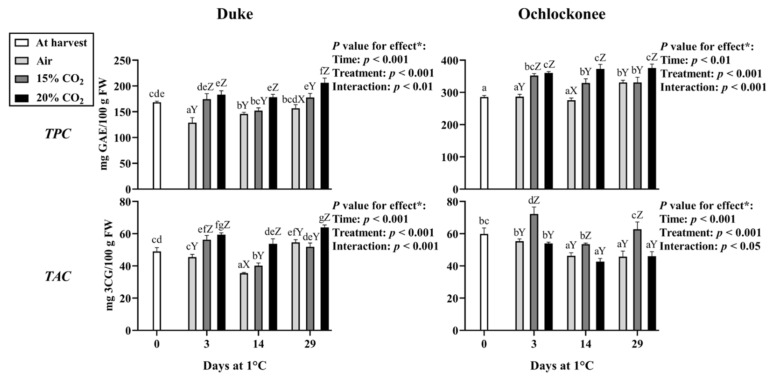
Total phenolic content (TPC) and total anthocyanin content (TAC) in Duke and Ochlockonee blueberries under short-term CO_2_ treatments during cold storage. Values represent mean ± SD (*n* = 6). Different lowercase letters indicate significant differences among all groups, while different uppercase letters indicate significant differences among treatments within the same day (Tukey’s test, *p* < 0.05). (*) *p*-values for storage time, treatment, and their interaction are shown in each panel.

In Duke blueberries, total phenolic content (TPC; Figure 4) decreased in non-treated fruit during storage, and by day 29, it reached values comparable to those at harvest (day 0). In contrast, TPC was generally maintained in CO_2_-treated samples. At day 14, only 20% CO_2_-treated fruit showed significantly higher values compared with non-treated fruit. A significant increase in TPC was observed in fruit treated with 15 and 20% CO_2_ in most sampling points. However, these values should be interpreted as global estimates of total reducing capacity rather than compound-specific concentrations, as determined by the Folin–Ciocalteu assay.

In Ochlockonee, TPC increased early at day 3 in response to both CO_2_ treatments and remained stable throughout storage, whereas non-treated fruit showed a significant increase only by day 29. In general, the highest TPC levels were observed in 20% CO_2_-treated Ochlockonee fruit.

Total anthocyanin content (TAC; Figure 4) in Duke followed a similar trend to that observed for TPC, with a decline in non-treated fruit until day 29. In contrast, both CO_2_ treatments induced a transient rise at day 3, sustained only in fruit treated with 20% CO_2_, resulting in the highest values at days 14 and 29 compared to non-treated and 15% CO_2_-treated fruit.

In Ochlockonee, fruit treated with 15% CO_2_ showed a significant increase in TAC at day 3 followed by a decrease, whereas 20% CO_2_-treated and non-treated fruit maintained lower and relatively stable levels. Moreover, 15% CO_2_ fruit showed the highest TAC values at all sampling points compared with non-treated or 20% CO_2_-treated samples. A strong positive correlation between TPC and TAC was observed in Duke (r = 0.774, *p* < 0.01), but not in Ochlockonee (Supplementary Tables S2 and S3).

It should be noted that total anthocyanin content represents a bulk spectrophotometric estimate of the anthocyanin pool and does not reflect individual anthocyanin species resolved by HPLC-QTOF analysis.

### 3.3. Effect of Short-Term CO_2_ Treatment on the Antioxidant Capacity

**Figure 5:**
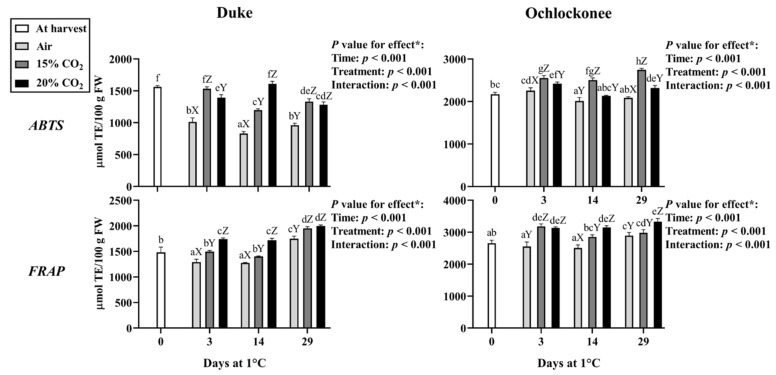
Antioxidant activity determined by ABTS and FRAP assays, in Duke and Ochlockonee blueberries under short-term CO_2_ treatments during cold storage. Values represent mean ± SD (*n* = 6). Different lowercase letters indicate significant differences among all groups, while different uppercase letters indicate significant differences among treatments within the same day (Tukey’s test, *p* < 0.05). (*) *p*-values for storage time, treatment, and their interaction are shown in each panel.

In Duke blueberries, antioxidant capacity measured by ABTS decreased early (day 3) in non-treated fruit and remained low throughout storage (Figure 5), whereas treated fruit remained at levels similar to or slightly below those observed at harvest. Overall, at each sampling point, CO_2_-treated fruit showed higher ABTS values than non-treated fruit. FRAP assays showed a different pattern (Figure 5): non-treated fruit declined during storage, while 15% CO_2_-treated fruit remained relatively stable, and both treatments increased by day 29. However, 20% CO_2_-treated fruit showed an early increase from day 3, converging with that of 15% CO_2_-treated fruit by day 29. Across all sampling points, fruit treated with 20% CO_2_ generally exhibited the highest FRAP values.

In Ochlockonee, ABTS antioxidant capacity was stable or slightly decreased at day 14 in non-treated fruit but increased overall in CO_2_-treated samples. The highest ABTS values were consistently observed in fruit treated with 15% CO_2_ across all sampling points. FRAP results showed a similar trend, although in this case, non-treated fruit remained stable and increased at day 29, whereas treated fruit showed a progressive increase during storage. At most sampling points, 20% CO_2_-treated fruit exhibited the highest FRAP values (Figure 5).

In Duke, statistical analysis showed that FRAP and ABTS assays were not significantly correlated with each other. However, both antioxidant measures were significantly and positively correlated with total phenolic content (FRAP: r = 0.788; ABTS: r = 0.577, *p* < 0.01) and total anthocyanin content (FRAP: r = 0.764; ABTS: r = 0.506, *p* < 0.01). In Ochlockonee, FRAP and ABTS were significantly positively correlated with each other (r = 0.382, *p* < 0.05), and both showed significant positive correlations with total phenolic content (FRAP: r = 0.915, *p* < 0.01) and total anthocyanin content (ABTS: r = 0.647, *p* < 0.01) (Supplementary Tables S2 and S3).

These results reflect changes in antioxidant capacity as measured by chemical assays and should be interpreted as indicative of variations in redox-related metabolites rather than direct evidence of in vivo oxidative status or stress mitigation.

### 3.4. Effect of Short-Term CO_2_ Treatment on the Metabolic Profile

In order to characterize the phenolic profile of CO_2_-treated and non-treated highbush and rabbiteye blueberries, a targeted metabolomic analysis was performed using HPLC/QTOF-MS under both positive and negative ionization modes. Table 1 shows the fragmentation patterns and identification of several compounds, based on reference data from the Phenol-Explorer database.

A total of 39 phenolic compounds were putatively identified based on their mass spectra, fragmentation patterns, and comparison with previously reported data under the chromatographic and MS conditions used in this study. Of these, 18 were detected in positive ionization mode and 21 in negative mode. The identified compounds included 12 anthocyanins, seven flavonols, four flavan-3-ols/proanthocyanidins, and seven phenolic acids. One compound corresponded to a less common flavonol, and six signals were assigned as putative isomers of previously described compounds, which could not be unequivocally distinguished under the present analytical conditions.

Additional minor peaks were detected; however, they were not included due to insufficient fragmentation data for reliable structural annotation. Therefore, only compounds with consistent MS/MS evidence were reported to ensure analytical robustness. It should be noted that compounds are reported with different levels of identification confidence (MSI levels 1–3), which should be considered when interpreting individual metabolite annotations.

**Figure 6:**
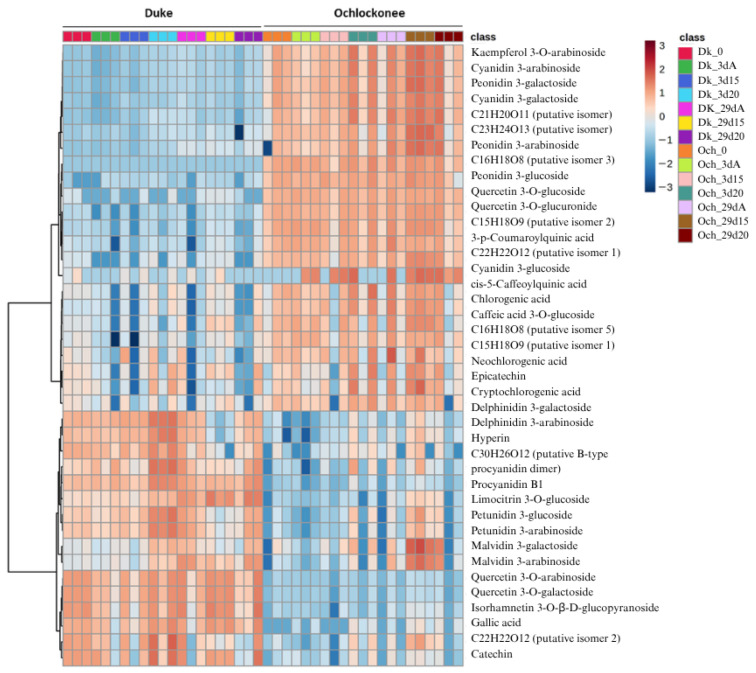
Heatmap of phenolic compounds in Duke and Ochlockonee blueberries under short-term CO_2_ treatments during cold storage. Rows represent individual polyphenols, and columns correspond to samples. Data were normalized and scaled prior to analysis. Hierarchical clustering was performed using Euclidean distance and Ward’s method. The color gradient indicates relative concentration, with red denoting higher levels and blue lower levels.

When clustered heatmap analysis was performed, compounds were primarily grouped according to blueberry genotype rather than the applied treatment (Figure 6), indicating that genotype was the main factor driving the overall metabolomic variation. This pattern reflects inherent genotype-associated differences together with preharvest conditions that define the baseline metabolic composition prior to storage. However, this observation should be interpreted as a multivariate trend rather than evidence of a dominant effect of a single factor.

Interestingly, the compounds found in higher concentrations in Duke blueberries, mainly anthocyanins, phenolic acids, flavonols, one flavanol, and several unidentified isomers, were present in lower amounts in Ochlockonee fruit. Conversely, the compounds that were less abundant in Duke, including anthocyanins, proanthocyanidins, flavonols, one phenolic acid, and one flavanol, showed higher concentrations in Ochlockonee.

In relation to the short-term gaseous treatments applied to Duke blueberries, treatment-associated changes were observed for specific metabolites, including an increase in certain anthocyanins, such as delphinidin-3-galactoside, delphinidin-3-arabinoside, petunidin-3-arabinoside, petunidin-3-glucoside, as well as the flavan-3-ol catechin, after 3 days of exposure to 20% CO_2_. At the end of storage, Duke fruit treated with 15% CO_2_ exhibited high levels of flavonols such as quercetin-3-arabinoside, quercetin-3-galatoside and isorhamnetin, as well as the flavan-3-ol catechin and the phenolic acid gallic acid. Moreover, the flavonol limocetrin showed a gradual increase over the storage period, reaching its highest levels on day 29, with slightly higher concentrations in both treated fruit.

In Ochlockonee blueberries, the flavonol kaempferol-3-arabinoside and anthocyanins such as cyanidin-3-arabinoside, peonidin-3-galactoside, cyanidin-3-galactoside, peonidin-3-arabinoside, cyanidin-3-glucoside, peonidin-3-glucoside, malvidin-3-galactoside and malvidin-3-arabinoside were more abundant in samples treated with 15% CO_2_ at the end of storage. Specifically, delphinidin-3-galactoside, delphinidin-3-arabinoside, petunidin-3-glucoside, petunidin-3-arabinoside, malvidin-3-galactoside and malvidin-3-arabinoside, together with the flavonol limocetrin, showed increased abundance in 15% CO_2_-treated Ochlockonee fruit, with malvidin-3-galactoside and malvidin-3-arabinoside showing the most pronounced response.

Correlation analysis between gene expression and individual phenolic compounds identified by HPLC-QTOF profiling revealed genotype-associated patterns. In Duke, *PAL* showed the most consistent positive associations with specific anthocyanins, including peonidin-3-arabinoside and malvidin-3-arabinoside (*p* < 0.01), peonidin-3-galactoside (*p* < 0.05), and was the only gene significantly correlated with cyanidin-3-galactoside, cyanidin-3-arabinoside, and malvidin-3-galactoside (*p* < 0.05). In contrast, *CHS* did not show significant correlations with any of the analyzed metabolites. In Ochlockonee, significant associations were more limited, mainly involving *PAL* and cyanidin-3-glucoside (*p* < 0.05).

Overall, these results indicate that while CO_2_ treatments are associated with changes in specific phenolic compounds, the global metabolomic profile is primarily shaped by genotype, with treatment effects acting as a secondary modulation of a pre-existing genotype-determined metabolic background.

### 3.5. Differential Multivariate Responses of Blueberry Cultivars to Short-Term CO_2_ and Cold Storage Revealed by PCA

**Figure 7:**
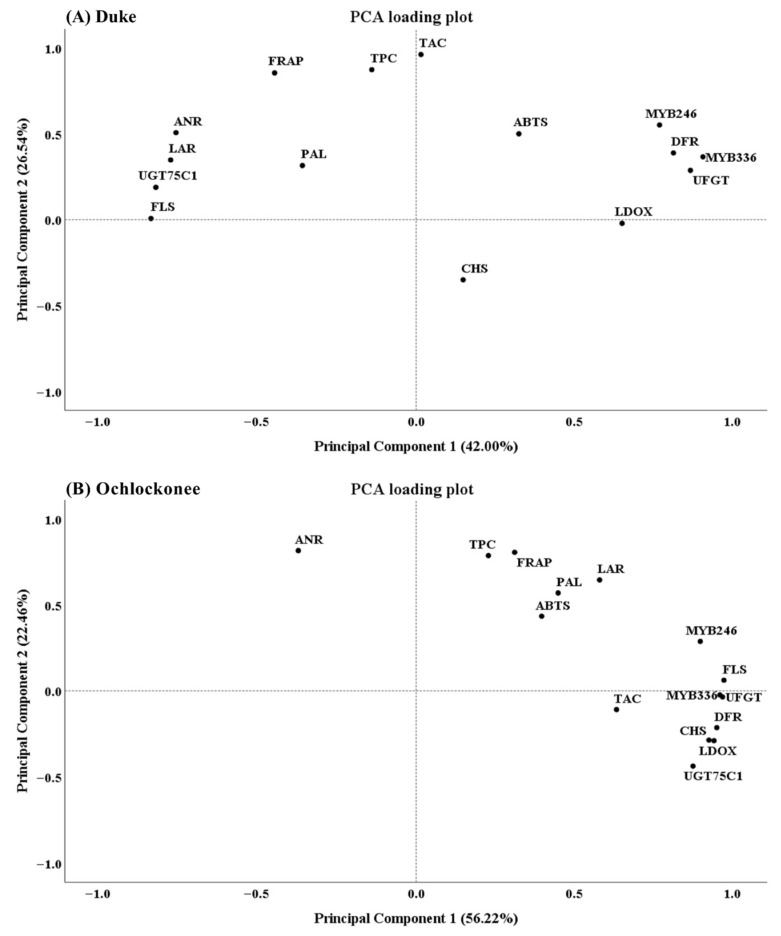
Principal component analysis (PCA) of phenolic compounds, antioxidant capacity, and gene expression data in (**A**) Duke and (**B**) Ochlockonee blueberries under short-term CO_2_ treatments during cold storage. Each point represents the mean of three biological replicates obtained from independent pooled fruit samples per treatment and sampling time. Data were standardized prior to analysis.

PCA revealed a clear separation between antioxidant-related traits and gene expression variables in the Duke cultivar (Figure 7A). Along component 1, which accounted for 51.88% of the variance, most structural and regulatory genes associated with flavonoid and anthocyanin biosynthesis (*DFR*, *UFGT*, *LDOX*, *MYB246* and *MYB336*) were located on the positive side, alongside ABTS. Conversely, several genes associated with the phenylpropanoid pathway (*FLS*, *LAR*, *ANR* and *UGT75C1*) were positioned on the negative side of component 1, indicating a different distribution pattern within the pathway. *PAL* was situated near the origin, suggesting a limited contribution to the explained variance. Along component 2, which explained 24.06% of the variance, TPC and TAC were closely clustered at high positive values, indicating a close association in their distribution. Meanwhile, FRAP was located in the upper-left quadrant, partially separated from ABTS. Notably, *CHS* was distinctly separated by negative component 2 values, suggesting a distinct distribution pattern relative to the other biosynthetic genes.

In the Ochlockonee cultivar, PCA also revealed a strong structuring of the variables, albeit with a different distribution pattern (Figure 7B). Component 1, accounting for 56.22% of the variance, was characterized by a clear clustering of anthocyanin-related genes (*CHS*, *DFR*, *UFGT*, *FLS*, *UGT75C1*, *LDOX*, *MYB246* and *MYB336*) together with TAC on the positive side, indicating a close distribution of these variables in the multivariate space. In contrast, *ANR* was located on the negative side of component 1, showing an opposite trend to the main gene cluster. Along component 2, which accounted for 22.46% of the variance, the antioxidant-related variables were clearly separated. TPC and FRAP were grouped at high positive values, while ABTS was associated with *PAL* and *LAR* in the upper-right quadrant. These variables were distinct from the TAC-gene cluster, suggesting that general antioxidant capacity is partially separated from anthocyanin-related variables in this analysis.

Taken together, the PCA suggests that gene expression variables and antioxidant-related parameters show different distribution patterns in both cultivars, although the relationships among variables vary between them. In Duke, ABTS was positioned closer to several gene expression variables, whereas TPC and TAC formed a separate cluster. In contrast, in Ochlockonee, anthocyanin-related variables and gene expression showed a closer distribution, while antioxidant-related variables were more clearly separated. These patterns should be interpreted as exploratory associations rather than evidence of direct functional relationships.

## 4. Discussion

The growing demand for blueberries, driven in part by their recognized health benefits [38], has increased the need for efficient postharvest strategies to maintain fruit quality. Short-term treatments with high concentrations of CO_2_ have been reported to preserve berry quality during storage, including in strawberries and raspberries, where they are associated with delayed decay and maintenance of firmness [23,39]. In blueberries, applying 15% or 20% CO_2_ for 3 days at 1 °C preserved the quality of the two blueberry species analyzed in this study for up to 29 days and improved firmness in the highbush Duke cultivar [24].

While these effects are well documented at the physiological and technological level, their underlying metabolic and transcriptional bases remain less clearly characterized. In this context, the present study provides an integrated analysis of phenolic composition, antioxidant-related traits, and gene expression in two contrasting blueberry cultivars (Duke and Ochlockonee), representing different species and harvest periods. However, it should be noted that the experimental design compares two cultivar/season combinations, reflecting the objective of assessing CO_2_ treatment effects in blueberries harvested at the commercial maturity stage, rather than establishing species-wide responses. In addition, because fruits were harvested according to their natural production cycles (June vs. September), preharvest environmental conditions may have influenced the observed differences, as light, temperature, and developmental stage strongly affect phenolic metabolism in fruit [40]. Therefore, the responses described here likely reflect the combined influence of genotype, preharvest conditions, and postharvest treatment.

Short-term high-CO_2_ treatments were associated with changes in the expression of phenylpropanoid-related genes, particularly those involved in anthocyanin biosynthesis and their regulatory *MYB* transcription factors (Supplementary Figures S1 and S2). In Duke, *DFR*, *LDOX*, *UFGT*, and *MYBs* showed transient induction, declining during storage except for *LDOX*. In contrast, Ochlockonee exhibited a broader transcriptional response involving early pathway genes (*PAL*, *CHS*), as well as branch-specific genes, suggesting a more extensive but still transient activation of the phenylpropanoid pathway.

Genes related to flavan-3-ol (*ANR*, *LAR*) and flavonol biosynthesis (*FLS*) displayed genotype-dependent patterns, indicating differential modulation of pathway branches by CO_2_ across cultivars. The consistent increase in *ANR* transcript levels, particularly under 20% CO_2_, may indicate a differential modulation of this branch of the pathway under the applied conditions, in agreement with observations in table grapes where CO_2_ treatments affected flavanol-related genes [41]. This suggests a potential role of *ANR* in stress-associated flavonoid responses, although further evidence would be required to confirm its conservation across species. In contrast, *LAR* showed a biphasic and genotype-associated expression pattern, indicating that its regulation is influenced by both storage duration and atmosphere composition.

*FLS* expression further highlighted contrasting regulatory strategies. In Duke, expression gradually increased toward the end of storage, which may reflect differences in flavonol-related metabolic responses during storage. Ochlockonee, however, exhibited a rapid but transient induction after CO_2_ treatment, showing a similar temporal pattern to anthocyanin-related genes.

*MYB* transcription factors showed genotype-associated patterns with phenylpropanoid genes. In Duke, these associations were mainly restricted to anthocyanin-related genes, whereas in Ochlockonee they extended across a broader set of flavonoid pathway genes, suggesting a more integrated regulatory network. This pattern was further supported by correlation analysis, where in Duke, *MYB246* and *MYB336* were significantly associated primarily with key anthocyanin biosynthetic genes (*DFR*, *LDOX*, and *UFGT*) and with each other, whereas in Ochlockonee, *MYB* transcription factors correlated with a wider set of phenylpropanoid-related genes, with the exception of *ANR*. These findings are consistent with previous reports describing the role of R2R3-MYB transcription factors in anthocyanin regulation in blueberries [27] and with studies in other fruit species reporting postharvest modulation of flavonoid-related genes [41]. However, in the present study, these associations should be interpreted as correlative, as no protein-level, enzymatic, or metabolic flux data were obtained, and therefore, regulatory relationships between *MYB*s and downstream genes require further validation.

Together, these genotype-associated responses suggest that short-term high-CO_2_ treatments are associated with anthocyanin-related transcriptional changes, which may contribute to the modulation of secondary metabolism during cold storage, although this cannot be directly inferred from the present data. Consistent with previous studies, *PAL* has been reported as responsive to cold storage in several species, and flavonoid-associated gene expression changes under cold conditions have been described in *Actinidia arguta* [42] and *Arabidopsis thaliana* [43]. Similar findings have been reported in table grapes, where short-term CO_2_ treatments have been associated with enhanced flavonoid-related gene expression and improved antioxidant retention [41].

Changes in total phenolic and anthocyanin contents indicate that short-term high-CO_2_ treatments are associated with the maintenance or, in some cases, an increase in secondary metabolite levels during cold storage. In Duke, 20% CO_2_ notably increased the content of both compounds, while in Ochlockonee, total phenolics rose under both treatments, but only 15% CO_2_ effectively maintained anthocyanins. These results are consistent with a potential role of CO_2_ treatments in maintaining aspects of blueberry nutritional quality during cold storage, in line with previous findings in rabbiteye cultivars [21].

Interestingly, the antioxidant capacity, as determined by ABTS and FRAP assays, showed genotype-specific correlations with phenolic and anthocyanin content. In Duke, both assays correlated with phenolics and anthocyanins, while in Ochlockonee, ABTS was primarily associated with anthocyanins and FRAP with phenolics, which may reflect methodological differences between the assays. Together, these relationships suggest that CO_2_ treatments are associated with changes in antioxidant-related parameters, consistent with reports in grapes and raspberries where short-term CO_2_ treatments have been shown to reduce or delay cold stress responses during storage [23,24,41]. However, as no direct measurements of oxidative stress or ROS metabolism were performed in this study, the potential role of stress alleviation remains to be confirmed.

HPLC-QTOF profiling revealed clear genotype-specific phenolic signatures across 39 identified compounds (anthocyanins, flavonols, proanthocyanidins, and phenolic acids) suggesting that genotype is a major factor shaping phenolic composition under the conditions studied, although other factors could also contribute. In this context, CO_2_ treatment effects may act upon a pre-established metabolic background determined by genotype and preharvest conditions, rather than inducing global metabolomic restructuring.

This interpretation is supported by clustering results, where samples were grouped primarily by genotype rather than treatment, indicating that baseline metabolic differences dominate the multivariate structure.

Some metabolites exhibited inverse abundance between cultivars, suggesting that Duke and Ochlockonee may differentially regulate metabolic pathways under the same CO_2_ conditions. These differences likely arise from developmental and physiological contrasts: Duke is an early-season highbush cultivar adapted to cooler climates (800–1000 chilling hours), while Ochlockonee is a late-season rabbiteye cultivar adapted to warmer conditions (400–600 chilling hours), which may affect metabolite profiles via light, temperature, and maturity cues. Seasonal factors such as light intensity, temperature, and physiological maturity could further modulate plant secondary metabolites [40], contributing to the observed species-specific responses to CO_2_ and cold storage. Comparable genotype-driven metabolite variation has been documented in raspberries [44] and sweet cherry cultivars [45].

In Duke blueberries, the early accumulation of metabolites such as delphinidin-3-glucoside, petunidin-3-arabinoside, and catechin after just 3 days at 20% CO_2_ indicated early changes in phenolic profiles, although less pronounced than in Ochlockonee. A similar increase in catechin was reported in strawberries treated with 20% CO_2_ for 3 days [22]. The progressive rise in limocetrin during storage, especially in treated fruit, may be associated with a sustained-related metabolic response, although this was not directly evaluated. In contrast, the specific behavior of malvidin derivatives in Ochlockonee under 15% CO_2_ highlights their potential as indicators associated with CO_2_ treatment response, consistent with findings in grapes showing increased flavonol and quercetin derivatives after CO_2_ treatment [46]. These results highlight that CO_2_-induced changes occur within a genotype-associated metabolic framework, emphasizing the importance of considering both genotype and preharvest background when interpreting postharvest metabolomic responses.

PCA revealed distinct multivariate distribution patterns between cultivars, reflecting differences in the coordination between transcriptional and antioxidant-related variables. While Duke showed a closer association between antioxidant capacity (particularly ABTS) and gene expression variables, Ochlockonee exhibited a tighter distribution between anthocyanin-related traits and gene expression, with antioxidant parameters appearing more independent. These contrasting profiles suggest differences in the distribution patterns of transcriptional and metabolic responses under the studied conditions. However, given the exploratory nature of PCA, these associations should be interpreted with caution.

From a practical and commercial perspective, short-term high-CO_2_ treatments (15–20%) applied for 3 days at low temperature could be readily integrated into existing cold chain systems used in commercial berry handling, as gaseous treatments can be implemented in current processing facilities and cold-storage chambers with minimal infrastructure modifications. Moreover, these approaches reduce concerns about chemical residues, since gases such as CO_2_ decompose without leaving harmful by-products, aligning with increasing consumer demand for safer and more sustainable produce [47]. Additionally, the short duration of these treatments may offer cost advantages compared to long-term CA storage, as modified conditions are only required for a limited period. Such treatments have been extensively investigated at the laboratory scale in berry postharvest systems, particularly in strawberries and raspberries, where short-term high CO_2_ treatments have consistently been shown to extend shelf life and reduce decay, supporting their technological feasibility [22,23,39]. However, their performance under commercial conditions remains to be fully established. Therefore, successful implementation in blueberries would require precise control of CO_2_ exposure to avoid potential off-flavor development or physiological stress responses, as well as validation under large-scale commercial conditions across different supply chains.

Overall, while the present results support the feasibility of CO_2_ as a short-term postharvest tool, further industrial validation is required before widespread adoption.

In this context, the results of this study indicate that short-term high-CO_2_ treatments are associated with transient modulation of phenylpropanoid metabolism during cold storage, affecting both gene expression and metabolite accumulation in a genotype-associated manner. These responses diminished during prolonged storage and were not associated with stable metabolic reprogramming. Therefore, no statistical comparisons between genotypes were performed, and observed differences should be interpreted as descriptive trends within a commercial production context.

## Figures and Tables

**Table 1:** Fragmentation patterns (MS/MS) and identification of several compounds from Duke and Ochlockonee blueberries, based on reference data from the Phenol-Explorer database, using both positive and negative ionization modes.

FooDB ID	Compound Name	MSI Level *	Formula	Monoisotopic Mass	RT (min)	Mode	MS/MS Fragments
FDB017159	Cyanidin 3-galactoside	2	C_21_H_21_O_11_	449.1084	11.53	POS	449.1017; 356.7234; 287.0531; 191.1007; 109.0274
FDB017314	Petunidin 3-arabinoside	2	C_21_H_21_O_11_	449.1084	14.99	POS	450.1175; 449.1070; 392.0165; 317.0165; 317.0648; 302.0347; 276.9331
FDB002603	Cyanidin 3-glucoside	1	C_21_H_21_O_11_	449.1084	12.62	POS	449.1066; 395.5965; 287.0504; 267.0701; 219.8052; 172.1356
	C_21_H_20_O_11_ (putative isomer)	3	C_21_H_20_O_11_	448.1006	11.07	POS	449.1053; 287.0525; 317.0447; 116.0557
FDB017148	Cyanidin 3-arabinoside	2	C_20_H_19_O_10_	419.0978	13.67	POS	419.0900; 360.1124; 287.0530; 245.0431; 155.0520
FDB017304	Peonidin 3-arabinoside	2	C_21_H_21_O_10_	433.1135	17.30	POS	433.1148; 338.8506; 301.0701; 286.0156; 272.9434; 259.6767
FDB017192	Delphinidin 3-arabinoside	2	C_20_H_19_O_11_	435.0927	11.04	POS	435.0417; 419.5752; 303.0469; 287.0799; 229.5420; 195.6830
FDB017307	Peonidin 3-galactoside	2	C_22_H_23_O_11_	463.1240	15.05	POS	463.1229; 327.7995; 301.0683; 286.0365; 258.0457; 163.3198
FDB017308	Peonidin 3-glucoside	2	C_22_H_23_O_11_	463.1240	16.27	POS	463.1030; 386.7232; 339.8765; 301.0745; 286.0230; 248.9436
FDB017210	Malvidin 3-arabinoside	2	C_22_H_23_O_11_	463.1240	18.12	POS	463.1226; 394.2657; 331.0784; 315.0420; 287.0436
FDB017202	Delphinidin 3-galactoside	2	C_21_H_21_O_12_	465.1033	9.29	POS	465.0762; 303.0426; 274.0449; 227.6956; 167.3231
FDB017317	Petunidin 3-glucoside	2	C_22_H_23_O_12_	479.1190	12.81	POS	479.1177; 437.7712; 356.8832; 317.0660; 302.0383; 274.8782; 238.3507
FDB017212	Malvidin 3-galactoside	2	C_23_H_25_O_12_	493.1346	15.99	POS	493.1328; 420.3294; 331.0798; 287.0564; 239.9218; 151.6816
FDB002714	Malvidin 3-glucoside	1	C_23_H_25_O_12_	493.1346	16.90	POS	493.1336; 415.7369; 331.0807; 226.8620
FDB000958	Procyanidin B1	2	C_30_H_26_O_12_	578.1424	9.04	POS	579.1400; 409.0879; 289.0736; 275.0422; 139.0361; 127.0375
FDB012206	C_30_H_26_O_12_ (putative B-type procyanidin dimer)	2	C_30_H_26_O_12_	578.1424	13.80	POS	579.1360; 409.0869; 287.0489; 139.0396; 127.0383
FDB000636	Kaempferol 3-O-arabinoside	2	C_20_H_18_O_10_	418.0900	13.68	POS	419.2957; 295.7844; 287.0551; 269.8651; 240.7465
FDB000653	Limocitrin 3-glucoside	2	C_23_H_24_O_13_	508.1217	27.26	POS	509.2713; 347.0717; 357.3055; 291.0847; 167.0319
	C_23_H_24_O_13_ (putative isomer)	3	C_23_H_24_O_13_	508.1217	27.46	POS	509.3079; 347.0738; 181.0549; 164.6608
FDB000662	Gallic acid	1	C_7_H_6_O_5_	170.0215	3.05	NEG	169.0137; 136.4318; 125.0186; 109.0483; 81.0248
FDB002571	Catechin	2	C_15_H_14_O_6_	290.0790	10.65	NEG	289.0785; 245.0828; 221.0749; 203.0644; 151.0377; 137.0188; 125.0162; 109.0304
FDB017126	Epicatechin	1	C_15_H_14_O_6_	290.0790	15.42	NEG	289.0885; 245.0891; 221.0915; 205.0527; 189.0403; 163.0361; 125.0195
FDB007443	Caffeic acid 3-glucoside	2	C_15_H_18_O_9_	342.0951	7.47	NEG	341.0993; 179.0362; 158.9705; 135.0447
FDB007443	C_15_H_18_O_9_ (putative isomer 1)	3	C_15_H_18_O_9_	342.0951	8.15	NEG	341.0940; 179.0391; 161.0248; 135.0337
FDB007443	C_15_H_18_O_9_ (putative isomer 2)	3	C_15_H_18_O_9_	342.0951	10.36	NEG	341.0986; 179.0336; 158.9319; 135.0437
FDB002562	Neochlorogenic acid	2	C_16_H_18_O_9_	354.0951	7.09	NEG	353.0992; 215.3132; 191.0594; 157.1754
FDB002582	Chlorogenic acid	1	C_16_H_18_O_9_	354.0951	11.00	NEG	353.0998; 251.0521; 191.0585; 161.0301; 109.0275
FDB002561	Cryptochlorogenic acid	2	C_16_H_18_O_9_	354.0951	12.10	NEG	353.0940; 262.8409; 191.0598; 179.0328; 161.0208; 135.0400
FDB000275	Cis-5-Caffeoylquinic acid	2	C_16_H_18_O_9_	354.0951	14.98	NEG	353.1001; 303.5892; 191.0589; 127.0325; 77.9300

* Identification confidence levels were assigned following the Metabolomics Standards Initiative (MSI) criteria [37]. MSI level 1: identified compounds confirmed with authentic standards analyzed under the same experimental conditions; MSI level 2: putatively annotated compounds based on MS/MS fragmentation data and comparison with databases and/or literature; MSI level 3: putatively characterized compounds or unresolved isomers.

## Data Availability

The original contributions presented in this study are included in the article/Supplementary Material. Further inquiries can be directed to the corresponding author.

## Supplementary Materials

The following supporting information can be downloaded at: https://www.mdpi.com/article/10.3390/plants15101496/s1. The supporting information includes Tables S1–S3 (list of primer sequences and correlation analyses) and Figures S1 and S2 (gene expression heatmaps).

## References

[1] Pap N., Fidelis M., Azevedo L., do Carmo M.A.V., Wang D., Mocan A., Pereira E.P.R., Xavier-Santos D., Sant’Ana A.S., Yang B. (2021). Berry polyphenols and human health: Evidence of antioxidant, anti-inflammatory, microbiota modulation, and cell-protecting effects. Curr. Opin. Food Sci..

[2] Cerezo A.B., Cătunescu G.M., González M.M.P., Hornedo-Ortega R., Pop C.R., Rusu C.C., Chirilă F., Rotar A.M., Garcia-Parrilla M.C., Troncoso A.M. (2020). Anthocyanins in blueberries grown in hot climate exert strong antioxidant activity and may be effective against urinary tract bacteria. Antioxidants.

[3] Macha Huamán R., Navarro Soto F.C., Ramírez Ríos A., Alfaro Paredes E.A. (2023). International market concentration of fresh blueberries in the period 2001–2020. Humanit. Soc. Sci. Commun..

[4] Kader A.A. (2002). Postharvest Technology of Horticultural Crops.

[5] Ghosh A., Debnath S.C., Igamberdiev A.U. (2024). Effects of Vaccinium-derived antioxidants on human health: The past, present and future. Front. Mol. Biosci..

[6] Biała W., Jasiński M. (2018). The phenylpropanoid case–It is transport that matters. Front. Plant Sci..

[7] Zheng Y., Wang C.Y., Wang S.Y., Zheng W. (2003). Effect of high-oxygen atmospheres on blueberry phenolics, anthocyanins, and antioxidant capacity. J. Agric. Food Chem..

[8] Magri A., Petriccione M. (2022). Melatonin treatment reduces qualitative decay and improves antioxidant system in highbush blueberry fruit during cold storage. J. Sci. Food Agric..

[9] Fanourakis D., Makraki T., Spyrou G.P., Karavidas I., Tsaniklidis G., Ntatsi G. (2025). Environmental Drivers of Fruit Quality and Shelf Life in Greenhouse Vegetables: Species-Specific Insights. Agronomy.

[10] Ortiz A., Sansinenea E. (2023). Phenylpropanoid derivatives and their role in plants’ health and as antimicrobials. Curr. Microbiol..

[11] Liu J., Osbourn A., Ma P. (2015). MYB transcription factors as regulators of phenylpropanoid metabolism in plants. Mol. Plant.

[12] Bogs J., Downey M.O., Harvey J.S., Ashton A.R., Tanner G.J., Robinson S.P. (2005). Proanthocyanidin synthesis and expression of genes encoding *leucoanthocyanidin reductase* and anthocyanidin reductase in developing grape berries and grapevine leaves. Plant Physiol..

[13] Ben-Simhon Z., Judeinstein S., Trainin T., Harel-Beja R., Bar-Yaakov I., Borochov-Neori H., Holland D. (2015). A “white” anthocyanin-less pomegranate (*Punica granatum* L.) caused by an insertion in the coding region of the leucoanthocyanidin dioxygenase (*LDOX*; *ANS*) gene. PLoS ONE.

[14] Ma D., Constabel C.P. (2019). MYB repressors as regulators of phenylpropanoid metabolism in plants. Trends Plant Sci..

[15] Zhang X., He Y., Li L., Liu H., Hong G. (2021). Involvement of the R2R3-MYB transcription factor MYB21 and its homologs in regulating flavonol accumulation in Arabidopsis stamen. J. Exp. Bot..

[16] de Pascual-Teresa S., Sanchez-Ballesta M.T. (2008). Anthocyanins: From plant to health. Phytochem. Rev..

[17] Horvitz S. (2017). Postharvest handling of berries. Postharvest Handling.

[18] Connor A.M., Luby J.J., Hancock J.F., Berkheimer S., Hanson E.J. (2002). Changes in fruit antioxidant activity among blueberry cultivars during cold-temperature storage. J. Agric. Food Chem..

[19] Castrejón A.D.R., Eichholz I., Rohn S., Kroh L.W., Huyskens-Keil S. (2008). Phenolic profile and antioxidant activity of highbush blueberry (*Vaccinium corymbosum* L.) during fruit maturation and ripening. Food Chem..

[20] Sanchez-Ballesta M.T., Marti-Anders C., Álvarez M.D., Escribano M.I., Merodio C., Romero I. (2023). Are the blueberries we buy good quality? Comparative study of berries purchased from different outlets. Foods.

[21] Gao H., Hu W., Jiang A., Zhou F., Guan Y., Feng K., Gaowa S. (2021). Effects of high CO_2_ on the quality and antioxidant capacity of postharvest blueberries (*Vaccinium* spp.). J. Food Meas. Charact..

[22] Blanch M., Alvarez I., Sanchez-Ballesta M.T., Escribano M.I., Merodio C. (2012). Increasing catechin and procyanidin accumulation in high-CO_2_-treated *Fragaria vesca* strawberries. J. Agric. Food Chem..

[23] Romero I., Toledo-Guerrero J.D., Álvarez M.D., Herranz B., Escribano M.I., Merodio C., Sanchez-Ballesta M.T. (2025). Short-term gaseous treatments preserve firmness and fruit quality in raspberries stored at low temperature: Impact on the expression of cell wall remodeling genes. Postharvest Biol. Technol..

[24] Toledo-Guerrero J.D., Álvarez M.D., Herranz B., Escribano M.I., Merodio C., Romero I., Sanchez-Ballesta M.T. (2024). Effect of short-term high-CO_2_ treatments on the quality of highbush and rabbiteye blueberries during cold storage. Plants.

[25] Beaudry R. (1992). Blueberry quality characteristics and how they can be optimized. Ann. Rep. Mich. State Hort. Soc..

[26] Yu D., Tang H., Zhang Y., Du Z., Yu H., Chen Q. (2012). Comparison and improvement of different methods of RNA isolation from strawberry (*Fragaria × ananassa*). J. Agric. Sci..

[27] Wang H., Zhai L., Wang S., Zheng B., Hu H., Li X., Bian S. (2023). Identification of R2R3-MYB family in blueberry and its potential involvement of anthocyanin biosynthesis in fruits. BMC Genomics.

[28] Untergasser A., Cutcutache I., Koressaar T., Ye J., Faircloth B.C., Remm M., Rozen S.G. (2012). Primer3—New capabilities and interfaces. Nucleic Acids Res..

[29] Livak K.J., Schmittgen T.D. (2001). Analysis of Relative Gene Expression Data Using Real-Time Quantitative PCR and the 2^−ΔΔCT^ Method. Methods.

[30] Deng Y., Li Y., Sun H. (2020). Selection of reference genes for RT qPCR normalization in blueberry (*Vaccinium corymbosum* × *angustifolium*) under various abiotic stresses. FEBS Open Bio.

[31] Singleton V.L., Rossi J.A. (1965). Colorimetry of total phenolics with phosphomolybdic-phosphotungstic acid reagents. Am. J. Enol. Vitic..

[32] Wrolstad R.E. (1993). Color and Pigment Analyses in Fruit Products.

[33] Sanchez-Ballesta M.T., Romero I., Jiménez J.B., Orea J.M., González-Ureña A., Escribano M.I., Merodio C. (2007). Involvement of phenylpropanoid pathway in the response of table grapes to low temperature and high CO_2_ levels. Postharvest Biol. Technol..

[34] Re R., Pellegrini N., Proteggente A., Pannala A., Yang M., Rice-Evans C. (1999). Antioxidant activity applying an improved ABTS radical cation decolorization assay. Free Radic. Biol. Med..

[35] Benzie I.F.F., Strain J.J. (1996). The Ferric Reducing Ability of Plasma (FRAP) as a measure of “antioxidant power”: The FRAP assay. Anal. Biochem..

[36] Sanchez-Ballesta M.T., Balderas C., Escribano M.I., Merodio C., Romero I. (2025). Metabolic and antioxidant variations in “Regina” raspberries: A comparative analysis of early and late harvests. Plants.

[37] Sumner L.W., Amberg A., Barrett D., Beale M.H., Beger R., Daykin C.A., Fan T.W.-M., Fiehn O., Goodacre R., Griffin J.L. (2007). Proposed minimum reporting standards for chemical analysis. Metabolomics.

[38] Silva S., Costa E.M., Veiga M., Morais R.M., Calhau C., Pintado M. (2020). Health promoting properties of blueberries: A review. Crit. Rev. Food Sci. Nutr..

[39] Eum H.L., Han S.H., Lee E.J. (2021). High-CO_2_ treatment prolongs the postharvest shelf life of strawberry fruits by reducing decay and cell wall degradation. Foods.

[40] Qaderi M.M., Martel A.B., Strugnell C.A. (2023). Environmental factors regulate plant secondary metabolites. Plants.

[41] Romero I., Domínguez I., Morales-Diaz N., Escribano M.I., Merodio C., Sanchez-Ballesta M.T. (2020). Regulation of flavonoid biosynthesis pathway by a single or dual short-term CO_2_ treatment in black table grapes stored at low temperature. Plant Physiol. Biochem..

[42] Sun S., Fang J., Lin M., Hu C., Qi X., Chen J., Zhong Y., Muhammad A., Li Z., Li Y. (2021). Comparative metabolomic and transcriptomic studies reveal key metabolism pathways contributing to freezing tolerance under cold stress in kiwifruit. Front. Plant Sci..

[43] Zhu Z.P., Yu J.X., Liu F.F., Zhu D.W., Xiong A.S., Sun M. (2023). AeWRKY32 from okra regulates anthocyanin accumulation and cold tolerance in Arabidopsis. J. Plant Physiol..

[44] Krüger E., Dietrich H., Schöpplein E., Rasim S., Kürbel P. (2011). Cultivar, storage conditions and ripening effects on physical and chemical qualities of red raspberry fruit. Postharvest Biol. Technol..

[45] Commisso M., Bianconi M., Di Carlo F., Poletti S., Bulgarini A., Munari F., Negri S., Stocchero M., Ceoldo S., Avesani L. (2017). Multi-approach metabolomics analysis and artificial simplified phytocomplexes reveal cultivar-dependent synergy between polyphenols and ascorbic acid in fruits of the sweet cherry (*Prunus avium* L.). PLoS ONE.

[46] Maoz I., de Rosso M., Kaplunov T., Vedova A.D., Sela N., Flamini R., Lewinsohn E., Lichter A. (2019). Metabolomic and transcriptomic changes underlying cold and anaerobic stresses after storage of table grapes. Sci. Rep..

[47] Sandhya (2010). Modified atmosphere packaging of fresh produce: Current status and future needs. LWT Food Sci. Technol..

