# Supplementary material for: Short-Term High-CO2 Treatment Modulates Phenylpropanoid Metabolism and Antioxidant Capacity in Blueberries During Cold Storage
Source: Plants (Basel). 2026 May 14;15(10):1496. doi: 10.3390/plants15101496 (PMC13210411; doi:10.3390/plants15101496)
Supplement: Supplementary file 1 [file plants-15-01496-s001.zip › Supplementary Tables S2 and S3.pdf]

Table S2. Pearson correlation matrix showing the relationships between total phenolic content (TPC), total anthocyanin content (TAC), antioxidant capacity (ABTS and FRAP) and the expression levels of phenylpropanoid biosynthetic genes in Duke highbush blueberries. Negative correlations are shown in brown and positive correlations in green. Color intensity reflects the strength and significance of the correlations: darker shades indicate stronger and more statistically significant relationships.

	ABTS	FRAP	TPC	TAC	*PAL*	*CHS*	*FLS*	*DFR*	*LAR*	*LDOX*	*ANR*	*UFGT*	*UGT75C1*	*MYB336*	*MYB246*
**ABTS**	1	0,333	,577**	,506**	-,510**	-0,164	-0,178	0,215	-0,325	-0,058	-0,120	0,314	-0,303	0,351	,368*
**FRAP**	0,333	1	,788**	,764**	,389*	-0,285	,468**	-0,056	,582**	-0,233	,686**	-0,176	,597**	-0,080	0,162
**TPC**	,577**	,788**	1	,774**	-0,010	-,471**	0,185	0,081	0,177	-0,187	,508**	0,018	0,138	0,134	0,333
**TAC**	,506**	,764**	,774**	1	0,305	-0,146	-0,017	,381*	,378*	-0,022	,510**	0,337	0,134	0,353	,470**
PAL	-,510**	,389*	-0,010	0,305	1	0,024	0,291	0,094	,772**	0,152	,447*	-0,035	,551**	-0,098	0,019
CHS	-0,164	-0,285	-,471**	-0,146	0,024	1	0,028	0,082	-0,013	0,299	-0,262	0,274	0,039	0,089	-0,123
FLS	-0,178	,468**	0,185	-0,017	0,291	0,028	1	-,808**	,571**	-0,219	,507**	-,680**	,783**	-,740**	-,586**
DFR	0,215	-0,056	0,081	,381*	0,094	0,082	-,808**	1	-0,318	,463*	-0,346	,841**	-,512**	,908**	,854**
LAR	-0,325	,582**	0,177	,378*	,772**	-0,013	,571**	-0,318	1	-,381*	,831**	-,406*	,786**	-,522**	-,412*
LDOX	-0,058	-0,233	-0,187	-0,022	0,152	0,299	-0,219	,463*	-,381*	1	-,572**	,657**	-0,356	,627**	,592**
ANR	-0,120	,686**	,508**	,510**	,447*	-0,262	,507**	-0,346	,831**	-,572**	1	-,463**	,631**	-,489**	-0,336
UFGT	0,314	-0,176	0,018	0,337	-0,035	0,274	-,680**	,841**	-,406*	,657**	-,463**	1	-,539**	,907**	,769**
UGT75C1	-0,303	,597**	0,138	0,134	,551**	0,039	,783**	-,512**	,786**	-0,356	,631**	-,539**	1	-,592**	-,441*
MYB336	0,351	-0,080	0,134	0,353	-0,098	0,089	-,740**	,908**	-,522**	,627**	-,489**	,907**	-,592**	1	,933**
MYB246	,368*	0,162	0,333	,470**	0,019	-0,123	-,586**	,854**	-,412*	,592**	-0,336	,769**	-,441*	,933**	1

** Significant correlation at $p < 0.01$.

* Significant correlation at $p < 0.05$.

Table S3. Pearson correlation matrix showing the relationships between total phenolic content (TPC), total anthocyanin content (TAC), antioxidant capacity (ABTS and FRAP) and the expression levels of phenylpropanoid biosynthetic genes in Ochlockonee rabbiteye blueberries. Negative correlations are shown in brown and positive correlations in green. Color intensity reflects the strength and significance of the correlation: darker shades indicate stronger and more statistically significant relationships.

	ABTS	FRAP	TPC	TAC	PAL	CHS	FLS	DFR	LAR	LDOX	ANR	UFGT	UGT75C1	MYB336	MYB246
ABTS	1	,382*	0,330	,647**	,408*	0,150	0,308	0,191	,397*	0,149	0,087	,382*	0,117	0,280	,394*
FRAP	,382*	1	,915**	0,023	0,287	0,090	,388*	0,097	,519**	0,056	,411*	0,323	-0,024	0,236	,492**
TPC	0,330	,915**	1	-0,122	0,306	0,039	0,302	-0,011	,412*	-0,020	,366*	0,215	-0,075	0,179	,425*
TAC	,647**	0,023	-0,122	1	0,128	,452*	,449*	,600**	0,342	,536**	-0,292	,667**	,536**	,464**	,462*
PAL	,408*	0,287	0,306	0,128	1	0,234	,482**	0,278	,697**	0,255	0,349	0,270	0,102	,510**	,611**
CHS	0,150	0,090	0,039	,452*	0,234	1	,886**	,941**	0,338	,970**	-,568**	,882**	,941**	,896**	,714**
FLS	0,308	,388*	0,302	,449*	,482**	,886**	1	,887**	,546**	,891**	-0,325	,889**	,808**	,958**	,897**
DFR	0,191	0,097	-0,011	,600**	0,278	,941**	,887**	1	,505**	,979**	-,438*	,931**	,916**	,901**	,760**
LAR	,397*	,519**	,412*	0,342	,697**	0,338	,546**	,505**	1	,390*	,521**	,534**	0,204	,517**	,659**
LDOX	0,149	0,056	-0,020	,536**	0,255	,970**	,891**	,979**	,390*	1	-,546**	,897**	,955**	,906**	,740**
ANR	0,087	,411*	,366*	-0,292	0,349	-,568**	-0,325	-,438*	,521**	-,546**	1	-,380*	-,702**	-,390*	-0,139
UFGT	,382*	0,323	0,215	,667**	0,270	,882**	,889**	,931**	,534**	,897**	-,380*	1	,850**	,882**	,821**
UGT75C1	0,117	-0,024	-0,075	,536**	0,102	,941**	,808**	,916**	0,204	,955**	-,702**	,850**	1	,824**	,634**
MYB336	0,280	0,236	0,179	,464**	,510**	,896**	,958**	,901**	,517**	,906**	-,390*	,882**	,824**	1	,914**
MYB246	,394*	,492**	,425*	,462*	,611**	,714**	,897**	,760**	,659**	,740**	-0,139	,821**	,634**	,914**	1

** Significant correlation at $p < 0.01$.

* Significant correlation at $p < 0.05$.
