# Supplementary figures and images for: Short-Term High-CO2 Treatment Modulates Phenylpropanoid Metabolism and Antioxidant Capacity in Blueberries During Cold Storage
Source: Plants (Basel). 2026 May 14;15(10):1496. doi: 10.3390/plants15101496 (PMC13210411; doi:10.3390/plants15101496)

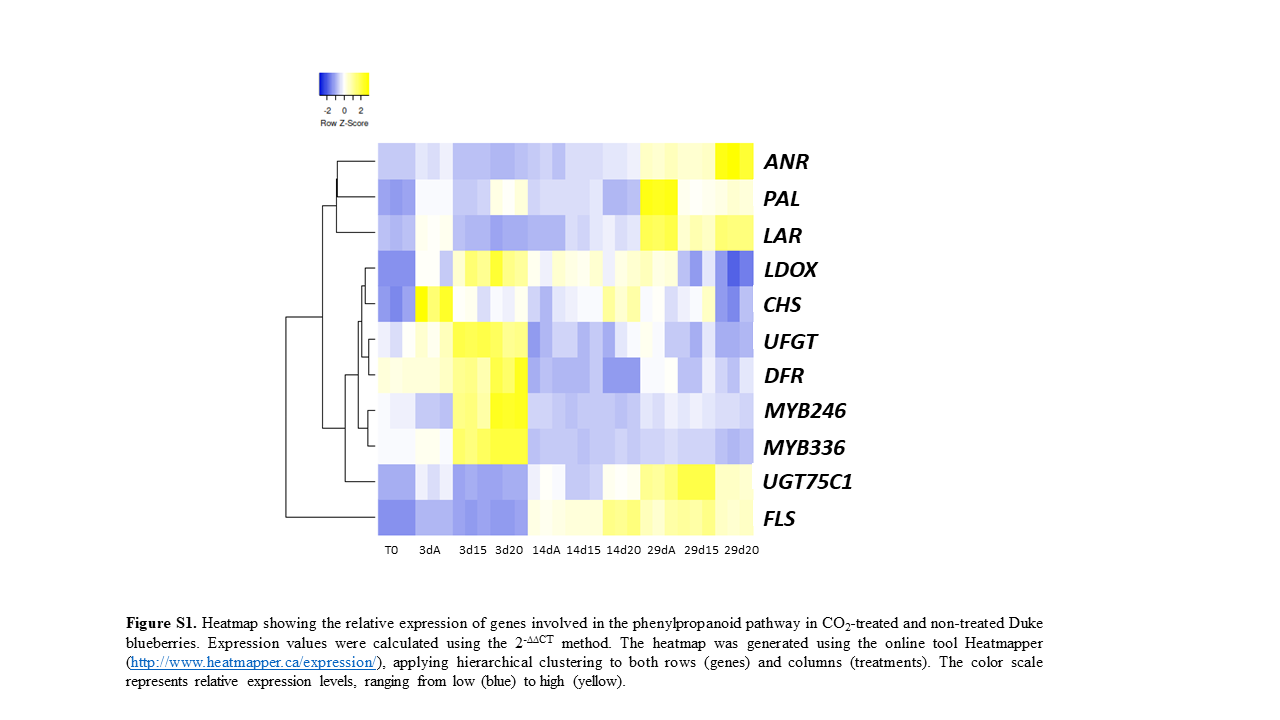

Supplement: Supplementary file 1 [file plants-15-01496-s001.zip › Supplementary Figure S1.tif]

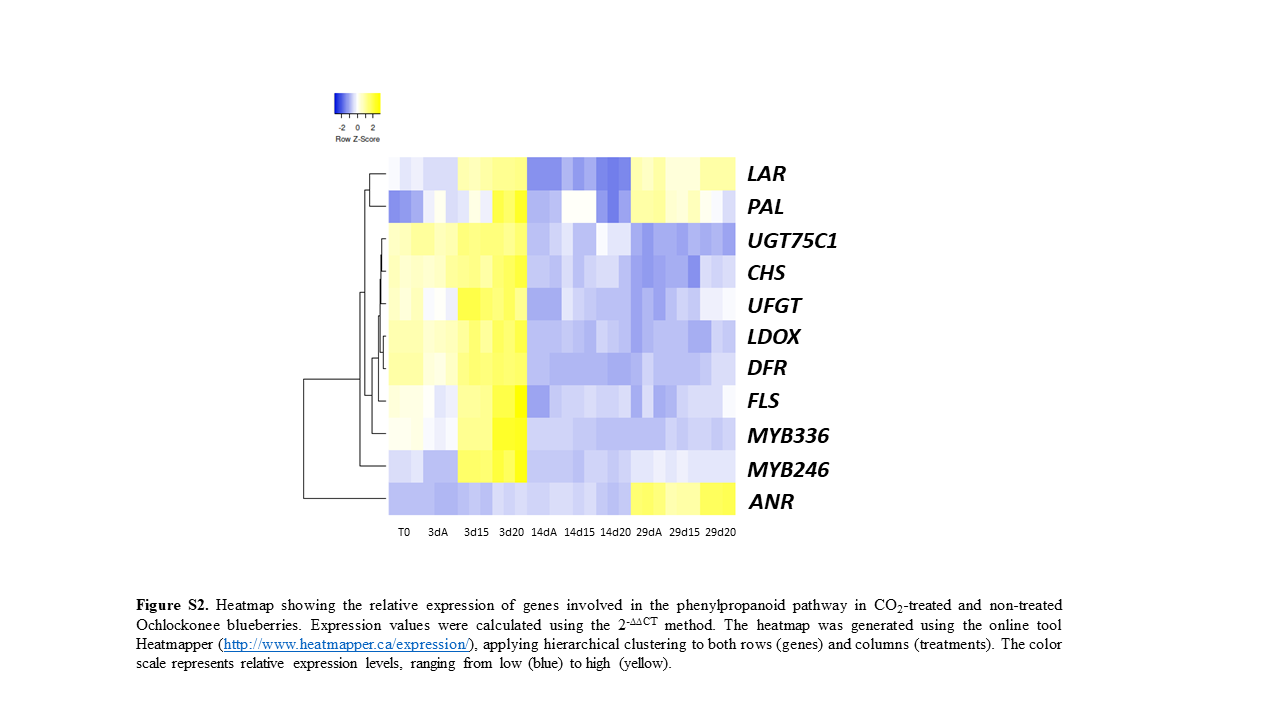

Supplement: Supplementary file 1 [file plants-15-01496-s001.zip › Supplementary Figure S2.tif]
